# RNA recombination: non-negligible factor for preventing emergence or reemergence of Senecavirus A

**DOI:** 10.3389/fvets.2024.1357179

**Published:** 2024-01-24

**Authors:** Yan Li, Tianyu Liu, Youming Zhang, Xiaoxiao Duan, Fuxiao Liu

**Affiliations:** ^1^College of Veterinary Medicine, Qingdao Agricultural University, Qingdao, China; ^2^Qingdao Center for Animal Disease Control and Prevention, Qingdao, China; ^3^State Key Laboratory of Microbial Technology, Shandong University, Qingdao, China

**Keywords:** Senecavirus A, RNA recombination, copy-choice recombination, evolution, infection

## Introduction

As an emerging virus, Senecavirus A (SVA) induces the vesicular disease in pigs. Clinical cases initially show mild signs characterized by lethargy and lameness, usually followed by the development of vesicles on the snout, dewclaw or (and) coronary band. SVA-elicited signs are generally indistinguishable from those of other vesicular diseases (1). In addition, SVA may cause epidemic transient neonatal losses in swine (2, 3). Although SVA infection was initially found at a Canada market in 2007 (4), the viral prototype had been recognized as a contaminant in culture of PER.C6 cells in 2002 (5). A retrospective study unexpectedly exhibited that SVA was circulating in the pig population of the United States as early as 1988, or even earlier (6). To date, SVA has been found in several countries, including Canada (4), the United States (7), Brazil (8), China (9), Thailand (10), Vietnam (11) and more recently Chile (12), therefore attracting a great deal of attention from the pig industry worldwide.

SVA belongs to the genus *Senecavirus* in the family *Picornaviridae*. Its virion is an icosahedral particle, approximately 30 nm in diameter. Its genome is a positive-sense, single-stranded and non-segmented RNA, approximately 7 300 nt in length, with a 3' poly(A) tail but without 5'-end capped structure. The genome contains 5' and 3' untranslated regions, and a long encoding region of polyprotein precursor. After SVA infection in a susceptible cell, the viral polyprotein precursor will be translated in the cytoplasm, and then progressively cleaved into 12 polypeptides, namely L, VP4, VP2, VP3, VP1, 2A, 2B, 2C, 3A, 3B, 3C and 3D (5). The VP1 to VP4 are four structural proteins. Their 60 copies form an icosahedral capsid with the typical architecture of picornavirus. The other proteins are non-structural proteins, involved in viral genome replication, host cell metabolism, and immune evasion (13). Mutual replication between genome and antigenome is catalyzed by a picornavirus-encoded RNA-dependent RNA polymerase (RdRp), also termed 3D polymerase (14).

## Selective pressure prompting SVA evolution

Understanding the origin and evolution of SVA is important for its prevention and surveillance. It is speculated that SVA originates from the United States in the 1980s, subsequently spreading to other countries and regions (15). To date, SVA has evolved into eight distinct lineages, including Clade Ancestor and Clade I to VII (16). Genomic differentiation progressively emerged with the continuous SVA evolution. Due to the low-fidelity characteristics of SVA RdRp, SVA displays a relatively high mutation rate during serial passaging *in vitro*, as demonstrated by our previous study (17). Therefore, SVA can rapidly enhance its own adaptive ability *via* synonymous codon bias evolution (18). Indeed, the codon preference analysis has indicated that natural selection is a primary driving force that affects the codon usage bias in SVA (15). Selection pressure analysis has additionally exhibited that the SVA polyprotein has been undergoing selection, with four amino acid residues located in the VP1, 2A, 3C, and 3D encoding regions that are under positive/diversifying selection (19).

## SVA intra-species recombination

Picornaviral members possess a typical feature, i.e., intra-species recombination, whereby viral progenies are produced from more than one parental genome (20). Such a recombination pattern can help picornaviruses adapt to a new environment, such as counteracting an error catastrophe within the viral genome (21). The intra-species recombination event has been demonstrated to occur in a population of picornaviruses, like enterovirus, aphthovirus and cardiovirus (22). Recently, it has been also reported that RNA recombination events have arisen among different SVA strains (23–29). Out of these reports, the earliest one characterized an SVA isolate (HeN-1/2018) from China in detail by the SimPlot analysis. The result revealed two breakpoints separating the viral genome into three regions, of which two fragments were independently derived from two genetically related variants of another country (23).

The intra-species recombination is an unpredictable event (30). Liu et al. (29) showed that two China variants (CH-GDZS-2019 and CH-GDMZ-2019), albeit isolated from the same province in 2019, had different results of recombination analysis. The CH-GDZS-2019 was genetically derived from USA-IA44662–2015-P1 and USA-GBI29-2015, both of which were isolated from the USA in 2015, while the CH-GDMZ-2019 was genetically derived from two China isolates (29). More recently, Wu et al. (27) collected a total of 238 SVA complete genomes from GenBank for recombination analysis by means of bioinformatic tools. The result showed that five isolates were identified as recombinants. Each of them was genetically derived from two or three isolates, which were suggested as the representatives of its putative parental lineages.

## Copy-choice recombination pattern

Intra-species recombination is a dominant genetic feature of picornaviruses, because these viruses exhibit remarkable structural or functional plasticity of their genomes (22). A typical model indicates that the picornaviral RdRp can switch a template during anti-genome (or genome) synthesis, causing occurrence of RNA recombination between two different individuals. Such a model was referred to as copy-choice recombination (20). The copy-choice recombination is responsible for the formation of recombinant RNA molecules through the RdRp switching from one template to another during genome/antigenome replication. For a given picornavirus, some special sequences are prone to occurrence of copy-choice recombination within them. These recombination-prone sequences are also known as recombination hotspots, which are associated both with RNA secondary structures and with nucleotide base composition (31). Determining recombination-prone sequences will uncover the existence of significant biases in the production of specific recombinant forms. Characterizing recombination-prone sequences will provide insight into a molecular mechanism involved in template switching (32). In addition, the RdRp plays an essential role in SVA replicative recombination, as evidenced by two RdRp variants (S460L and I212/S460L) that can reduce SVA recombination capacity (33).

As mentioned above, SVA has been continually evolving, and even recombining with one another (23–29). Unfortunately, the conclusion of recombination events was only deduced by the analysis using bioinformatic tools. We recently used reverse genetics technique to confirm experimentally that the copy-choice recombination could indeed occur inside a cell. We found that two lethal SVA cDNA clones, if independently transfected into cells, had no ability of virus recovery. However, their co-transfection led to the replication-competent virus successfully rescued from a cell monolayer. Sanger sequencing indicated the rescued virus with a wild-type genotype, implying that the copy-choice recombination event made the recombinant SVA avoid lethal mutations (34).

## Model of copy-choice recombination between SVAs

**Figure 1:**
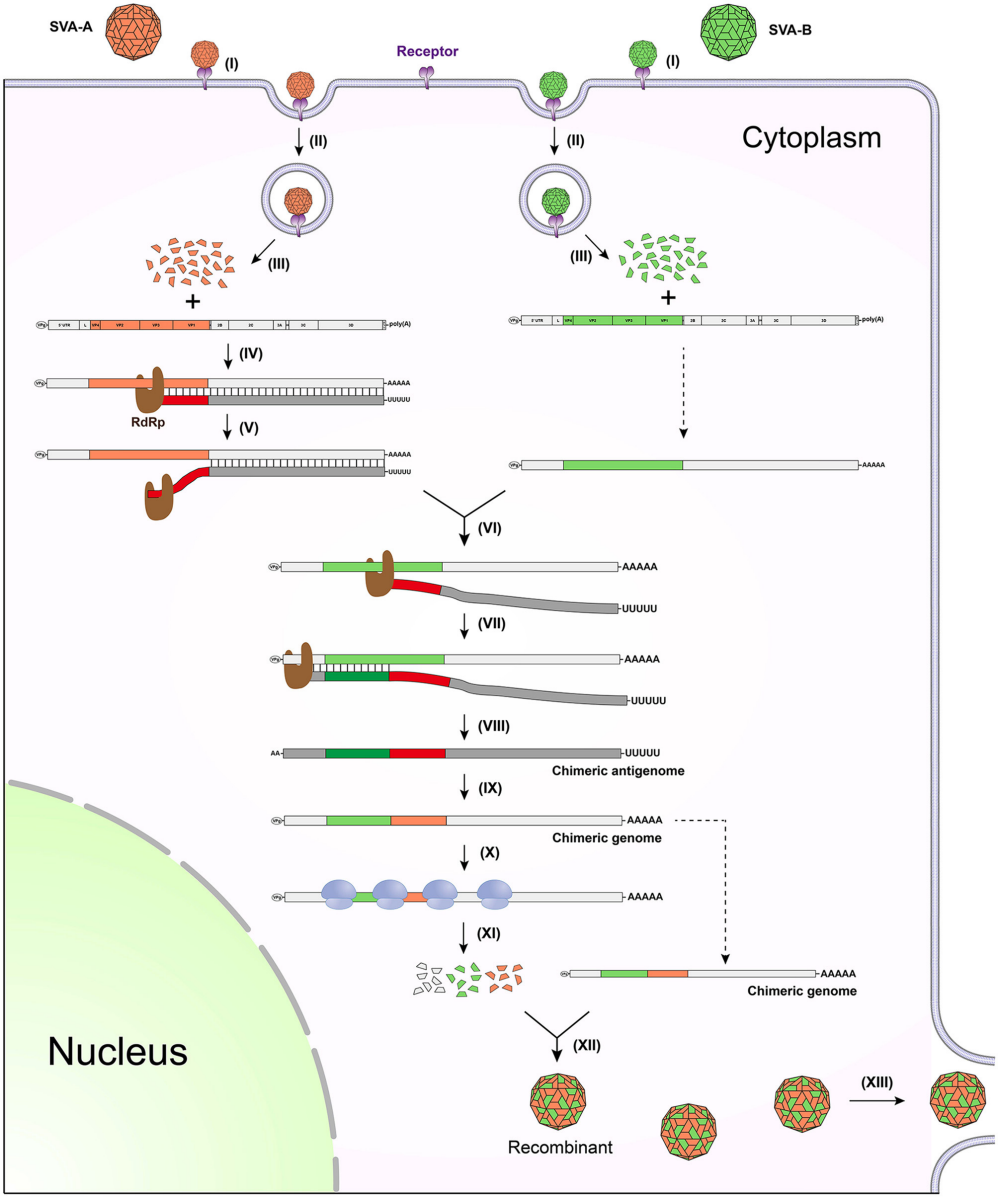
Schematic representation of copy-choice recombination event occurring between two different SVAs. (I) SVA-A and -B bind to receptors. (II) Viruses enter into the cell through endocytosis. (III) Viral genomes are released by uncoating of virions. (IV) RdRp mediates replication of antigenome. (V) RdRp detaches from the genome template. (VI) RdRp drags nascent incomplete antigenome to bind to the other genome template. (VII) RdRp continues to catalyze the synthesis of antigenome. (VIII) Nascent chimeric antigenome is released from the template. (IX) Chimeric genome is synthesized using the chimeric antigenome as template. (X) Ribosome binds to the chimeric genome for initiating translation. (XI) Viral proteins are expressed for further processing. (XII) The chimeric genome is encapsidated to generate a recombinant. (XIII) The recombinant is released from the cell.

If two different strains, SVA-A and -B, infect the same cell, copy-choice recombination may occur during genome/antigenome replication. Figure 1 schematically represents a series of events involved in how two different SVA virions infect a single cell, and more importantly how two genomes recombine with each other via the copy-choice pattern. In an SVA-A and -B-infected cell, the viral RdRp slides along the SVA-A genome for synthesizing its antigenome. If hindered by a physical barrier (e.g., a high-order RNA structure) during the replication of antigenome, the RdRp may detach from the genome template (Figure 1, step V), and then drag a nascent incomplete antigenome to search for an alternative template. The step VI briefly shows the process of template switching. The copy-choice recombination finally results in one recombinant that simultaneously harbors SVA-A and -B-derived sequences. If both SVA-A and -B harbor detrimental mutations but at two different positions in their genomes, the recombinant will “cleverly” evade these detrimental mutations, consequently achieving higher adaptability to a new environment.

## Copy-choice recombination improving virus fitness

Copy-choice recombination makes the progeny genome derive from more than one parental genome. This reproductive mode in virology is referred to as sexual replication (35), which can create considerable changes in the viral genome, allowing for antigenic shifts, host jumps, and fitness alteration. For example, copy-choice recombination of chikungunya virus can give rise to genome diversification, and even generate emerging variants that are positively selected in mosquitoes, thereby allowing chikungunya virus to overcome “tight” genetic bottlenecks or even providing an advantage in the improvement of viral fitness (36). The accumulation of mutations in viral RNA genomes perhaps leads to an error catastrophe, lethal to virus growth. However, poliovirus was demonstrated to be able of utilizing copy-choice recombination to evade the ribavirin-induced error catastrophe, therefore drastically enhancing its own fitness (21). Using defined imprecise recombinant viruses with Oxford Nanopore and Illumina next generation sequencing technologies, Bentley et al. (37) have drawn a conclusion that viruses undergo frequent and continuous recombination events over a prolonged period until the fittest viruses, predominantly those with wild-type length genomes, dominate the population.

## RNA recombination: non-negligible factor for preventing SVA infection

Although the emergence of viral variants is often involved in site mutations a given long-term selective pressure causes, both intra- and inter-species RNA recombination events would even directly cause the emergence or reemergence of positive-stranded RNA viruses. This is definitely a non-negligible issue for the prevention of emerging and reemerging diseases. As mentioned above, RNA recombination events continuously occur especially among intra-species individuals. For example, high recombination rates in the spike gene have been demonstrated to cause the emergence of lethal canine enteric coronaviruses (38). Similarly, porcine epidemic diarrhea virus has also revealed genetic changes in its spike gene, perhaps giving rise to the emergence of highly virulent variants in the field (39, 40). Enteroviruses, aphthoviruses and teschoviruses have shown phylogenetic segregation by serotype only in the structural region. Lack of segregation elsewhere has been proven to be attributable to extensive inter-serotype recombination (41).

Viral RNA recombination facilitates the ontogeny of viral variants with increased virulence and pathogenesis. The recombinant may resist immune responses, confer pathogenic effects in hosts, or (and) make itself be more transmissible in susceptible populations (30). Indeed, Bai et al. (2) showed that an SVA isolate from Shandong province in China was a putative recombinant, able to confer low fever, blisters, and lameness in pigs. More recently, another putative recombinant, also isolated from Shandong province, could elicit obvious clinical signs in pigs, and was capable of transmission to contact-exposed individuals (28). Therefore, the virulence enhancement is an adverse consequence SVA recombinants confer. This is the first non-negligible consequence resulting from the RNA recombination. Another key issue is a given SVA recombinant may be less virulent than its progenitor to hosts, causing the latent virus transmission in a population of pigs. If so, such a SVA recombinant would be a potential risk factor in hosts. It will gradually evolve along with common behavior of hosts, such as movement, mating and production. If there are changes in the herd environment, the SVA recombinant would possibly revert back to a status as highly virulent as that of its progenitor. This is the second non-negligible issue for preventing SVA infection. Last but not least, there is a potential risk, namely, RNA recombination that perhaps makes virus recombinants break through the host-range limitation. Although SVA infection is not regarded as zoonosis now, SVA has potent oncolytic activities in some human tumor cells (42). If SVA recombines with a human-derived virus, the emerging recombinant would have a potential in infecting humans, even inducing a severe zoonosis.

## Conclusions and future perspectives

Although SVA was found in the 1980s, how SVA originated was still unclear. SVA has been continually evolving via point mutations and RNA recombination. Compared with that of point mutations, the molecular mechanism of RNA recombination is greatly complicated. Moreover, compared with point mutations, although a map of RNA recombination can be represented by analysis using bioinformatic tools, the recombination process is not easily confirmed through an experiment. Therefore, RNA recombination among different SVAs is often neglected, consequently remaining a potential threat to the pig industry or even the public health. The pattern of copy-choice recombination makes SVA simultaneously acquire genomic sequences from two even more parental strains. This pattern randomly occurs in theory, and is unpredictable during the SVA evolution.

The outbreak of SVA infection was not frequently reported worldwide in recent 3 years. However, in order to prevent emergence or reemergence of SVA, RNA recombination should not be neglected by practitioners. Broad-spectrum antiviral agents should not be abused, because they possibly exert selection pressures on the SVA evolution. It is necessary now for taking action on continuous detection and surveillance for SVA recombination. Elucidation of the recombination-related mechanism will enable us to learn more about the relationship between RNA recombination and natural selection. Both epidemiological survey and phylogenetic analysis will contribute to the construction of a model for predicting major trends of SVA evolution in future.

## Author contributions

YL: Writing—original draft. TL: Writing—original draft. YZ: Writing—review & editing. XD: Writing—review & editing. FL: Writing—review & editing.
